# Volatiles from Cotton Plants Infested by *Agrotis segetum* (Lep.: Noctuidae) Attract the Larval Parasitoid *Microplitis mediator* (Hym.: Braconidae)

**DOI:** 10.3390/plants11070863

**Published:** 2022-03-24

**Authors:** Mengyu Li, Shike Xia, Tao Zhang, Livy Williams, Haijun Xiao, Yanhui Lu

**Affiliations:** 1 State Key Laboratory for Biology of Plant Diseases and Insect Pests, Institute of Plant Protection, Chinese Academy of Agricultural Sciences, Beijing 100193, China; limengyu19951111@163.com (M.L.); xiashikee@163.com (S.X.); 2 Institute of Entomology, Jiangxi Agricultural University, Nanchang 330045, China; 3 Integrated Pest Management Center of Hebei Province, Key Laboratory of IPM on Crops in Northern Region of North China, Institute of Plant Protection, Ministry of Agriculture, Hebei Academy of Agricultural and Forestry Sciences, Baoding 071000, China; cauzht@163.com; 4 USDA-ARS U.S. Vegetable Laboratory, Charleston, SC 29414, USA; livy.williams@usda.gov

**Keywords:** turnip moth, parasitoid wasp, herbivore-induced plant volatiles, behavioral response, biological control

## Abstract

Herbivore-induced plant volatiles (HIPVs), chemicals produced by plants infested by herbivorous insects, can act as kairomones that recruit natural enemies of the pest herbivore. *Agrotis segetum* (Denis and Schiffermüller) is a common, important pest of seedling cotton in Xinjiang Province, China, and the braconid *Microplitis mediator* (Haliday) is an important mortality factor of this pest’s larvae. In olfactometer tests, which included healthy foliage, infested foliage, or infested roots, *M. mediator* preferred *A. segetum*-infested cotton plants to healthy cotton plants. In GC-MS analyses of plant-emitted volatiles, we found that compounds emitted increased 14.9- and 13.3- fold after leaf infestation and root infestation, respectively, compared to healthy control plants. The volatiles were mainly p-xylene, nonanal, tetradecane, decanal, benzaldehyde, β-caryophyllene, and humulene, while linalool was only present in the leaf-infestation treatment. In addition, principal component analysis indicated that all 18 compounds were associated with the infested plants, especially β-caryophyllene, p-xylene, and decanal. Based on the above studies and previous functional evaluations of the volatile compounds, it can be demonstrated that these compounds play a crucial role in modulating the interactions between *A. segetum* and *M. mediator* and regulating parasitoid behavior. It may be possible to enhance the biological control of *A. segetum* by *M. mediator* through the application of HIPVs.

## 1. Introduction

Volatiles emitted from leaves, fruits, or flowers not only provide herbivorous insects with cues useful in foraging for nutritional resources, but also play an important role in herbivore oviposition behavior, host orientation, mate location, and mating behaviors [1,2,3]. For example, the preference of *Plutella xylostella* for cruciferous plants is due to the presence of isothiocyanates (ITCs), and these compounds also stimulate *P. xylostella* oviposition [4]. Fragrant volatiles emitted by flowers mediate the mirid bug *Apolygus lucorum*’s preference for flowering host plants [5], and temporal shifts in plant volatiles may regulate the host plant foraging behavior of *Ap. lucorum* adults [6]. Such flower-emitted volatile organic compounds (VOCs), when applied to fields, act as attractants for several mirids, including *Adelphocoris suturalis*, *Ad. lineolatus,* and *Ad. fasciaticollis* [7].

Plants damaged by herbivores also release herbivore-induced plant volatiles (HIPVs), such as terpenoids, green leaf volatiles (GLVs), and benzenoids [8]. HIPVs help the natural enemies of herbivores find their prey (or hosts) and mates, and they can also influence the foraging behavior of herbivores. For instance, adults of the predaceous lady beetle *Harmonia axyridis* showed a preference for aphid-infested plants over un-infested plants or aphids alone [9]. In addition, females of the parasitic wasp *Peristenus spretus* use *Ap. lucorum*-induced volatiles to locate hosts, and the parasitoid preference for flowering host plants was consistent with host preferences of *Ap. lucorum* [10]. The ability of HIPVs to increase the recruitment of natural enemies allows HIPVs to be used as natural enemy attractants in crop fields.

The cutworm known as the turnip moth, *Agrotis segetum* (Denis and Schiffermüller) (Lepidoptera: Noctuidae), is a significant underground pest that is widely distributed in China. It has a wide host range, including wheat, corn, cotton, and sugar beets. In the Xinjiang Province of China, *A. segetum* is a common, serious pest of seedling cotton [11] that can feed on the leaves, petioles, branches, and main stems of plants [12]. The larvae feed mainly near the ground on the stems of seedlings, causing severe crop loss and even the death of plants [12,13]. HIPVs emitted from cotton plants infested by *A. segetum* larvae significantly deter the oviposition of conspecific females [14].

*Microplitis mediator* (Haliday) (Hymenoptera: Braconidae) is a solitary endoparasitoid of noctuid and geometrid caterpillars in Europe and Asia [15]. Because of its biological and ecological traits, *M. mediator* has a broad potential for application in agricultural fields for pest control. Studies have shown that parasitism by *M. mediator* on the larvae of *A. segetum* can reach 37.8% under laboratory conditions [16]. Compounds such as (*3E*)-4,8-dimethyl-1,3,7-nonatriene and (*E*,*E*)-4,8,12-trimethyl-1,3,7,11-tridecatetraene, which are emitted by cotton seedlings damaged by chewing caterpillars or sucking bugs, are attractive to *M. mediator* [17]. *M. mediator* uses volatiles emitted by *Helicoverpa armigera*-damaged cotton for host location and foraging [15]. Therefore, it is likely that HIPVs also play a significant role in the biological control of *A. segetum* by *M. mediator* [15]. However, the tritrophic interactions among *A. segetum*, *M. mediator*, and cotton plants have not been studied.

In this study, we used a Y-tube olfactometer and GC-MS to assess the behavioral responses of *M. mediator* females to *A. segetum*-infested cotton and to the HIPVs produced by such feeding, with a view to further exploring the interactions among cotton, *A. segetum,* and *M. mediator*.

## 2. Results

### 2.1. Behavioral Responses to Larvae-Infested Cotton Plants

**Figure 1:**
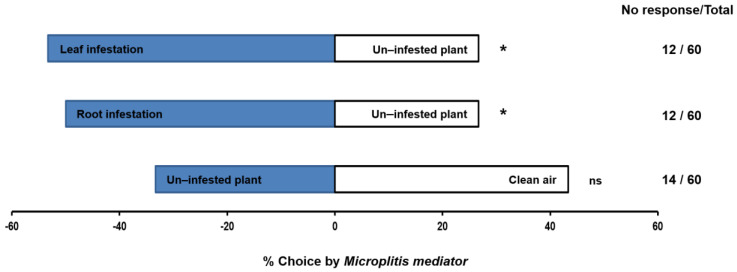
Behavioral responses of female *Microplitis mediator* adults to leaf and root volatiles induced by *Agrotis segetum* larval feeding. Female parasitoids had a choice between: (i) leaves infested by *A. segetum* larvae versus un-infested cotton plants, (ii) roots infested by *A. segetum* larvae versus un-infested cotton plants, and (iii) un-infested cotton plants versus clean air. “*” means a significant difference at the *p* < 0.05 level, while “ns” indicates no significant difference.

There were no significant differences between un-infested cotton plants and clean air, although a numerically greater proportion of female *M. mediator* (43.3%) chose clean air (*χ*^2^ = 0.78, df = 1, *p* = 0.3763). However, female parasitoids did prefer the odor from either *A. segetum*-infested leaves (*χ*^2^ = 5.33, df = 1, *p* = 0.0209) or *A. segetum*-infested roots (*χ*^2^ = 4.26, df = 1, *p* = 0.0390) over healthy, undamaged cotton plants (Figure 1).

### 2.2. Analysis of Cotton Volatiles

The cotton volatiles emitted from the three treatments (healthy leaves, infested leaves, and infested roots) differed (Table 1). Compounds often associated with air (e.g., toluene and benzene) or laboratory equipment (e.g., siloxanes or phthalates) were not included in our list of putative plant volatiles [18]. Compared to healthy plants, total emissions increased 14.9- and 13.3- fold after leaf and root infestation by *A. segetum* larvae, respectively. Of the 18 compounds detected, all but (1) β-caryophyllene, (2) γ-chlorobutyrophenone, (3) humulene, and (4) 5,9-undecadien-2-one, 6,10-dimethyl-, (E)- were emitted in significantly higher amounts from infested plants than from un-infested plants. The compound p-xylene had the greatest concentration, followed by nonanal, decanal, benzaldehyde, β-caryophyllene, and humulene. The concentrations of p-xylene, nonanal, and decanal were significantly higher from plants with pest-infested leaves, while tetradecane, β-caryophyllene, and humulene were released mainly by plants with pest-infested roots. Linalool was only present in plants with pest-infested leaves.

### 2.3. Principal Component Analysis

**Figure 2:**
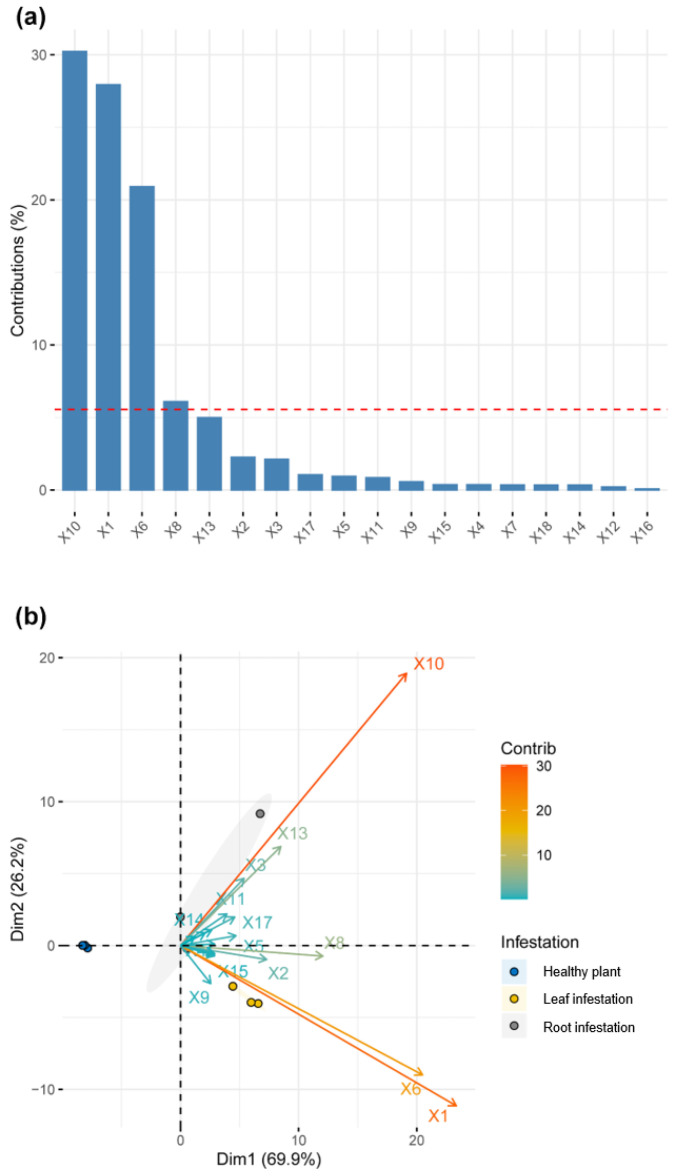
Principal component analysis (PCA) of volatile compounds induced by feeding of *Agrotis segetum* larvae on cotton plants. (**a**) Contribution of each volatile compound to the first two principal components (PC1 + PC2), which together explained >99% of all variation. The horizontal red dashed line represents the mean contribution (5.3%) of all 18 volatiles. (**b**) PCA biplot for assessing each volatile compound for three different treatments. The points with different colors represent samples from un-infested plants (blue), plants with larval feeding on leaves (yellow), and on roots (gray), respectively. Black lines with arrows indicate volatile compounds that were the top seven contributors to the first two PC-axes. X1: p-xylene; X2: nonanal; X3: tetradecane; X4: hexyl butyrate; X5: 1-hexanol, 2-ethyl-; X6: decanal; X7: pentadecane; X8: benzaldehyde; X9: linalool; X10: β-caryophyllene; X11: hexadecane; X12: γ-chlorobutyrophenone; X13: humulene; X14: heptadecane; X15: naphthalene; X16: 2-methylnaphthalene; X17: 5,9-undecadien-2-one, 6,10-dimethyl-, (E)-; X18: 1, 2-hexanediol.

PCA identified which volatile compounds dominated the volatile blends from healthy cotton plants or ones whose leaves or roots were infested by *A. segetum* larvae. Of the 18 components in the volatile blend, the major volatiles were β-caryophyllene, decanal, and p-xylene (Figure 2a), and all 18 compounds emitted from cotton plants contributed to the infestation process (Figure 2b).

## 3. Discussion

Volatile compounds play indispensable roles in the interactions among trophic levels in native food webs. HIPVs, as key information chemicals, provide herbivore-specific cues to parasitoids and predators [19]. We found that *M. mediator* was attracted by *A. segetum*-induced HIPVs. Li [14] showed that *A. segetum* females preferred to lay eggs on healthy (versus previously infested) cotton plants, and that the HIPVs induced by conspecific larvae on cotton plants had significant repellent effects on oviposition. Therefore, from an integrated pest management (IPM) perspective, leaf HIPVs in this system should both reduce *A. segetum* attacks and increase *M. mediator* parasitism of the pest larvae [14].

HIPVs can both repel herbivores and recruit natural enemies of pests [20]. However, comparative GC-MS analyses of headspace volatiles comparing compounds from healthy versus infested plants showed mostly quantitative, not qualitative effects. Linalool was the only compound that was present only in the VOCs of infested leaves. This compound is significantly repellent at high doses to the foraging and oviposition of several pest herbivores [14]. After infestation of a plant, the amount of p-xylene, nonanal, tetradecane, decanal, benzaldehyde, β-caryophyllene, and humulene all increased significantly. However, field and greenhouse experiments are necessary to confirm parasitoid attraction.

Insects can perceive chemical signals related to feeding, mating, and oviposition through diverse chemoreceptor families, including odorant receptors (ORs), and ionotropic receptors (IRs) [21,22,23]. For example, decanal was reported to be involved in the olfactory recognition process of *M. mediator* by binding strongly to MmedOBP18, which is mainly involved in the short-distance recognition of chemical information from hosts or host habitats [24]. Our results showed that the concentration of decanal emitted was significantly higher from plants with infested leaves (8.14 ± 0.48 μg/mL) compared to plants with infested roots (3.28 ± 0.66 μg/mL) or healthy (un-infested) plants (0.18 ± 0.05 μg/mL), suggesting that decanal plays an important role in the location of hosts and their habitats by *M. mediator*. In conclusion, plant volatiles induced by *A. segetum* can bind to both ORs and IRs, affecting both herbivores and their parasitoids (Table 2).

Currently, control of *A. segetum* in China relies on the application of chemical insecticides [12]. HIPVs play important roles in pest control in agriculture systems [35,36]. In our present study, female *M. mediator* wasps significantly preferred plants damaged by *A. segetum*, especially after foliar infestation. This preference may be related to changes in the release of HIPVs by the host plant. The amount of various volatile compounds in cotton increased after herbivore infestation, especially p-xylene, nonanal, tetradecane, decanal, benzaldehyde, β-caryophyllene, and humulene. Our results emphasize the important role of HIPVs in host selection by *M. mediator* and provide insights that may help improve the biological control of *A. segetum* through the combined application of HIPVs and the release of parasitic wasps. Future studies may explore parasitoid efficiency under laboratory and field conditions and investigate the effects of co-infestation of *H. armigera* and *A. segetum* on *M. mediator* in cotton.

## 4. Materials and Methods

### 4.1. Plants

Cotton (CCRI49) seeds were obtained from the Institute of Cotton Research of the Chinese Academy of Agricultural Sciences (CAAS) and sown in a greenhouse at Langfang Experimental Station, CAAS, under the following conditions: 26 ± 1 °C, 60 ± 10% RH, 14:10 h (L:D) photoperiod. Plants used for these tests were at the 3-true leaf growth stage.

### 4.2. Insects

*Agrotis segetum* larvae were reared continuously in a climate chamber under the same conditions described for plant production at the Langfang Experimental Station of CAAS. Second or third instar *A. segetum* larvae were used for our experiments.

The colony of *M. mediator* was established from diapausing cocoons provided by the Plant Protection Institute, Hebei Academy of Agriculture and Forestry Sciences. *M. mediator* larvae were reared in an incubator at 25 ± 1 °C, 60 ± 10% RH, 14:10 h (L:D) photoperiod at the Langfang Experimental Station, CAAS. All female parasitoids used in olfactometer tests were 3–6 d old, mated, and fed with 10% honey solution after emergence. Wasps had no previous oviposition experience or contact with plants before experiments.

### 4.3. Olfactometer Tests

A Y-tube olfactometer was used to evaluate the behavioral responses of 3–6 d old active, mated *M. mediator* adults (n = 60 females), when offered choices between the odors of (1) cotton leaves infested by four *A. segetum* larvae for 12 h, (2) cotton roots infested by four *A. segetum* larvae for 12 h, (3) un-infested plants, and (4) clean air. To ensure that *A. segetum* fed only on cotton leaves, we made a net bag (20 × 30 cm) with 120 mesh gauze to cover the above-ground parts of the cotton along with four individuals. In order to make *A. segetum* larvae feed only on cotton roots, we cut circular rings of blow molding paper (diam: 8 cm) to cover the cotton cotyledon stalks and fixed them with plastic rods, so that they could not climb higher. All parts of the equipment were connected with Teflon tubes, and the direction of air flow was from the atmospheric air intake, through activated charcoal, a distilled water humidification device, a gas flow control meter, a glass odor source vessel, and then into the Y-tube test arena, with similar parameters as those in previous studies [37].

One *M. mediator* adult was introduced to the initial test chamber after the airflow of both arms had been adjusted to 400 mL/min. Wasps that moved 1/3 of the way down a test arm within 5 min and stayed there for more than 10 s were counted as having made a ‘choice’, while wasps that did not respond as such were discarded and recorded as making ‘no choice’. After testing five parasitic wasps, the two arms of the Y-tube were reversed (with respect to their odor source), and after testing 10 wasps a clean Y-tube (washed with 95% ethanol and soaked and rinsed with distilled water and dried naturally at room temperature) was used.

### 4.4. Collection and Analysis of Cotton Volatiles

For volatile collection and identification of blends associated with our treatments, 3-true leaf cotton plants were separated into three groups: (1) leaves infested by four *A. segetum* larvae for 12 h; (2) roots infested by four *A. segetum* larvae for 12 h; (3) healthy, undamaged plants. To create infested plant foliage or roots, four larvae (second or third instar) of *A. segetum* (starved for 4 h) were placed on the whole plant and allowed to feed on foliage or roots for 12 h. Larvae were then removed, and the plants were immediately processed to collect headspace volatiles, which were then analyzed for their components.

Cotton headspace volatiles were collected from 1300 h to 1700 h every day using a dynamic headspace collection method. Cotton plants at the 3-true leaf stage (n = 3 for each treatment) were placed individually in a custom glass chamber (diam: 20 cm; height: 66 cm; Yuansu Glassware Supply Station, Shenzhen, China) and the soil was covered with aluminum foil before cotton volatile collection. An airflow at 500 mL/min passed over the plant, and volatiles were absorbed by 50 mg of Porapak^®^ Type Q adsorbent (Altech Assoc, Chicago, IL, USA). Then, cotton volatile samples were extracted into 1.5 mL sample bottles using 400 µL HPLC-grade n-Hexane (Aladdin, Shanghai, China) and, finally, were stored at −20 °C until GC-MS analyses.

GC-MS (GC: Agilent 7890A, equipped with a DB-WAX chromatographic column [30 m × 0.25 mm × 0.25 µm]; MS: Agilent 5975C) was used to analyze cotton volatiles, with similar parameters as those in previous studies [37]. The injector temperature for GC analysis was 230 °C, the oven temperature was kept at 50 °C for 1 min, and then raised by 5 °C/min to 180 °C for 2 min, and then increased by 10 °C/min to 230 °C, and held for 2 min. Helium was the carrier gas, at an average flow rate of 1 mL/min. The ion source temperature was 230 °C. The volatile compounds obtained were first identified by NIST 14 and were then compared with standard compounds to carry out qualitative-quantitative analyses.

### 4.5. Statistical Analysis

Chi-square tests were used to analyze the Y-tube olfactometer data to detect differences between the pairs of treatments. *χ*^2^ and P values were calculated, and non-responsive adults were excluded from the analysis. The amounts of each volatile compound emitted under different treatments were compared using one-way ANOVA, followed by Duncan’s new multiple range tests. Principal component analysis (PCA) was performed to analyze the patterns of volatiles from different treatments given its ability to reduce the complexity of the data while identifying the features in the dataset that contribute the most to the treatment effects. Chi-square tests and one-way ANOVAs were conducted using SPSS 25.0, while PCA analysis was performed using R 4.0.2 with a 0.05 level of significance.

## Figures and Tables

**Table 1:** Concentration of volatile compounds collected from cotton plants after infestation by *A. segetum* larvae.

Volatile Compound	Un-Infested Plants	Leaf Infestation	Root Infestation
p-Xylene	1.34 ± 0.22 c	10.59 ± 0.53 a	5.79 ± 0.35 b
Nonanal	0.22 ± 0.02 c	2.79 ± 0.07 a	1.84 ± 0.33 b
Tetradecane	0.14 ± 0.02 c	1.26 ± 0.08 b	2.90 ± 0.44 a
Hexyl butyrate	0.07 ± 0.02 b	1.13 ± 0.06 a	1.04 ± 0.13 a
1-Hexanol, 2-ethyl-	0.08 ± 0.01 b	1.53 ± 0.10 a	1.47 ± 0.25 a
Decanal	0.18 ± 0.05 c	8.14 ± 0.48 a	3.28 ± 0.66 b
Pentadecane	0.11 ± 0.03 b	1.07 ± 0.13 a	1.11 ± 0.20 a
Benzaldehyde	0.21 ± 0.003 b	4.29 ± 0.38 a	4.15 ± 0.25 a
Linalool	ND	1.23 ± 0.13	ND
β-caryophyllene	0.15 ± 0.02 a	3.79 ± 0.05 a	7.25 ± 3.34 a
Hexadecane	0.09 ± 0.02 b	1.11 ± 0.21 a	1.72 ± 0.32 a
γ-chlorobutyrophenone	0.07 ± 0.02 a	0.67 ± 0.05 a	0.66 ± 0.32 a
Humulene	0.07 ± 0.01 a	1.91 ± 0.08 a	3.10 ± 1.25 a
Heptadecane	0.09 ± 0.03 c	0.81 ± 0.16 b	1.49 ± 0.11 a
Naphthalene	0.06 ± 0.01 c	1.14 ± 0.10 a	0.88 ± 0.08 b
2-Methylnaphthalene	0.02 ± 0.01 c	0.33 ± 0.08 b	0.58 ± 0.07 a
5,9-Undecadien-2-one, 6,10-dimethyl-, (E)-	0.03 ± 0.00 a	1.41 ± 0.35 a	1.45 ± 0.62 a
1, 2-Hexanediol	0.04 ± 0.01 b	1.03 ± 0.05 a	0.83 ± 0.23 a
Total	2.97 ± 0.35	44.23 ± 1.62	39.54 ± 6.47

ND = not detected. Data are Mean ± SE (μg/mL); the same lowercase letters within rows indicate no significant difference based on ANOVA followed by Duncan’s MRT, α = 0.05.

**Table 2:** Known effects of host-plant volatiles on the behavioral activity and EAG activity of *Microplitis mediator* and their corresponding binding or recognition protein. The “+” indicates a substance inducing behavioral attraction or electrophysiological (EAG) activity in the antennae of *M. mediator* adults.

Volatile Compound	Behavioral Activity	EAG Activity	Corresponding Binding or Recognition Protein	Reference
p-Xylene	+	NT	MmedOBP8	[25,26]
Nonanal	+	+	MmedOBP8, MmedOBP9, MmedOBP10 MmedIR64a1, MmedIR64a2	[16,23,27,28]
Tetradecane	NT	NT	MmedIR64a1	[23]
Decanal	NT	+	MmedOBP18, MmedIR64a1, MmedIR64a2	[16,23,24,28]
Benzaldehyde	+	+	MmedOBP2, MmedCSP2, MmedNPC2a, MmedIR64a1	[16,23,25,29,30,31,32,33,34]
β-Caryophyllene	NT	+	MmedIR64a1	[23,32,33]
Humulene	NT	NT	MmedOBP4, MmedOBP6	[30]

## Data Availability

Not applicable.
